# PD128 Evaluating The Implementation Of An Abdominal Aortic Aneurysm Screening Program In Spain

**DOI:** 10.1017/S0266462324003659

**Published:** 2025-01-07

**Authors:** Anna Godo Pla, Gonzalo Bravo Soto, Antoni Sisó-Almirall, Melina Vega de Céniga, Marta Trapero Bertran, Ana Magdalena Vargas Martínez, Maria Dolors Estrada Sabadell, Rosa Maria Vivanco-Hidalgo

## Abstract

**Introduction:**

Abdominal aortic aneurysm (AAA) is a progressive and silent enlargement of the aorta, but aortic rupture is associated with a high mortality rate. The main objectives of this study were to assess evidence on the clinical safety, efficacy, and cost effectiveness of an AAA screening program using abdominal ultrasound. An economic evaluation was also conducted to assess the introduction of AAA screening into the Spanish Healthcare System.

**Methods:**

A systematic literature search was conducted according to PRISMA recommendations. Randomized controlled trials were selected to assess clinical safety and efficacy. Systematic reviews were selected to identify prognostic factors for AAA and full economic evaluations were selected for the cost-effectiveness analysis. Data extraction and assessment of evidence certainty were performed using the GRADE methodology. A Markov model was developed to perform an economic evaluation and budget impact analysis of an AAA screening program in the Spanish context.

**Results:**

Screening had a beneficial effect on AAA detection and rates of all-cause and AAA-related mortality in men older than 65 years. It had no effect on rates of AAA rupture or emergency surgery and may increase rates of elective surgery. The evidence was very uncertain for women older than 65 years. Sex and family history of AAA were the most important prognostic factors. Economic evaluation of a program screening men older than 65 years in primary care centers resulted in 0.33 life-years gained, 0.18 quality-adjusted life-years (QALYs) gained, and an incremental cost-effectiveness ratio of EUR81.98 per QALY. The program’s implementation would cost an additional EUR15.68 to EUR28.40 per patient.

**Conclusions:**

The implementation of an AAA screening program in men older than 65 years in Spain is considered effective and could be implemented using the infrastructure, materials, and human resources already present in the Spanish health system. Relevant indicators and dynamic monitoring of the program should be established to allow for continuous and flexible evaluation of results over time.

